# Improving Magnetofection of Magnetic Polyethylenimine Nanoparticles into MG-63 Osteoblasts Using a Novel Uniform Magnetic Field

**DOI:** 10.1186/s11671-019-2882-5

**Published:** 2019-03-12

**Authors:** Chaode Cen, Jun Wu, Yong Zhang, Cong Luo, Lina Xie, Xin Zhang, Xiaolan Yang, Ming Li, Yang Bi, Tingyu Li, Tongchuan He

**Affiliations:** 1 Department of Orthopedics, Guizhou Provincial Orthopedics Hospital, Guiyang, 550000 People’s Republic of China; 2 Department of Gynaecology, The First People’s Hospital of Guiyang, Guiyang, 550000 People’s Republic of China; 3 Department of Orthopedics, Laboratory of Orthopedic Biomaterials, Children’s Hospital of Chongqing Medical University, Chongqing, 400014 People’s Republic of China; 4 Ministry of Education Key Laboratory of Child Development and Disorders, Chongqing Engineering Research Center of Stem Cell Therapy, China International Science and Technology Cooperation base of Child Development and Critical Disorders, Chongqing, 400014 People’s Republic of China; 5 Ministry of Education Key Laboratory of Clinical Diagnostics, Department of Chemistry, Chongqing Medical University, Chongqing, 40016 People’s Republic of China; 6 Laboratory of Molecular Oncology, Department of Surgery/Orthopedics Center, The University of Chicago Medical Center, Chicago, IL 60637 USA

**Keywords:** Magnetofection, Magnetic nanoparticles, Uniform magnetic field, Polyethylenimine, Non-viral gene delivery

## Abstract

This study aimed to improve the magnetofection of MG-63 osteoblasts by integrating the use of a novel uniform magnetic field with low molecular weight polyethylenimine modified superparamagnetic iron oxide nanoparticles (PEI-SPIO-NPs). The excellent characteristics of PEI-SPIO-NPs such as size, zeta potential, the pDNA binding and protective ability were determined to be suitable for gene delivery. The novel uniform magnetic field enabled polyethylenimine-modified superparamagnetic iron oxide nanoparticles/pDNA complexes (PEI-SPIO-NPs/pDNA complexes) to rapidly and uniformly distribute on the surface of MG-63 cells, averting local transfection and decreasing disruption of the membrane caused by the centralization of positively charged PEI-SPIO-NPs, thereby increasing the effective coverage of magnetic gene carriers during transfection, and improving magnetofection efficiency. This innovative uniform magnetic field can be used to determine the optimal amount between PEI-SPIO-NPs and pDNA, as well as screen for the optimal formulation design of magnetic gene carrier under the homogenous conditions. Most importantly, the novel uniform magnetic field facilitates the transfection of PEI-SPIO-NPs/pDNA into osteoblasts, thereby providing a novel approach for the targeted delivery of therapeutic genes to osteosarcoma tissues as well as a reference for the treatment of other tumors.

## Background

Osteosarcoma is the most common malignant bone tumor that mainly affects children and adolescents. Because conventional therapies provide limited improvement, a new strategy for its treatment is imperative [1–3]. The advent of gene therapy has prompted researchers to assess its applications in osteosarcoma [4–6]. A safe and effective gene delivery system is critical to gene therapy. Gene delivery systems can be divided into viral gene delivery systems and non-viral gene delivery systems. Viral gene delivery systems have been shown to have high transfection efficiency in a variety of primary cells and cell lines. Viral gene delivery systems are strongly associated with safety issues such as triggering inflammatory responses and gene mutations [7], prompting researchers to divert their studies to non-viral gene delivery systems. However, most non-viral gene transfection techniques have not been particularly effective in transfecting osteosarcoma cell lines [8, 9].

Over the last decade, various physical methods such as gene guns [10], ultrasound [11], and electroporation [12] have been efficiently used in improving transfection. However, these physical methods also induce cell damage [13]. Because magnetic fields generally do not cause cellular damage, the technology of magnetic-assisted transfection has attracted the attention of researchers. Mah et al. introduced magnetic nanoparticles in gene transfection experiments by linking green fluorescent protein (GFP)-carrying recombinant adeno-associated viruses (rAAVs) to magnetic nanoparticles to achieve target-specific enhanced transfection both in vitro and in vivo. Compared with pure adenoviruses, magnetic adenovirus nanoparticles can reduce the dosage of the GFP-carrying rAAVs to 1% under the same transfection rate condition [14]. Scherer et al. coined the term “magnetofection” to denote magnet-mediated transfection using magnetic particles [15]. Subsequently, Plank et al. described the technique of magnetic transfection using non-viral vectors [16]. To further improve the magnetofection efficiency of non-viral vectors, Fouriki et al. adjusted the frequency and amplitude of an oscillating magnetic field to induce a dynamic mechanical stimulatory effect on magnetic nanoparticles, thereby increasing the probability of coming in contact with the target cells [17]. Oral et al. further improved transfection efficiency and reduced cytotoxicity of gene carriers by reversely rotating the hexagonal shaft of the magnet to control the speed and direction of the magnetic field [18]. In another study, Vainauska et al. used a dynamic gradient magnetic field that was generated by rotating a cylindrical permanent magnet for targeting delivery of magnetic gene carriers [19].

The efficiency of magnetofection has significantly improved as magnetic fields have evolved from being static and single to dynamic and sophisticated. However, studies focusing on the uniformity and effective range of the magnetic field are limited. To the best of our knowledge, most magnet devices cannot form uniform magnetic fields in a particular three-dimensional space, thereby resulting in heterogenous distribution of the magnetic nanoparticles, only achieving local transfection effectiveness of the magnetic field, and failing to address resulting cytotoxicities. Non-uniform magnetic field also hinders comparisons of magnetic transfection reagents as well as decrease transfection efficiency.

To resolve these problems, our research group in collaboration with the Electrical Engineering College of Chongqing University has developed a novel magnetic field generator [20, 21]. The magnetic field generator can form a uniform magnetic field at a certain height, and the added magnetic nanoparticles are rapidly and uniformly distributed at the bottom of the culture plate. The uniform magnetic field is not only convenient for comparative studies on the relationship between dose and transfection efficiency of various transfection reagents, but may also be utilized in assessing cellular uptake of magnetic nanoparticles.

In the present design, we aimed to establish a reliable non-viral gene delivery system with high magnetofection efficiency in osteosarcoma cell lines. The linear Mw20000 polyethylenimine (PEI-20000) was selected for the preparation of magnetic nanoparticles, because several previous studies have confirmed that linear PEI delivery systems have better transfection efficiency than branched PEI delivery systems in vivo [22, 23]. The properties of polyethylenimine modified superparamagnetic iron oxide nanoparticles (PEI-SPIO-NPs) were evaluated. A novel magnetic field generator was developed to form a uniform magnetic field, which was used to transfect PEI-SPIO-NPs/pDNA complexes into MG-63 osteosarcoma cell lines. The effects of different magnetic fields on the magnetofection were investigated systematically.

## Methods

### Materials and Reagents

Superparamagnetic iron oxide nanoparticles (SPIO-NPs), polyethylenimine hydrochloride (PEI), and Hoechst-33,324 were purchased from Sigma-Aldrich Co. (St Louis, MO, USA). PolyMag-200 (commercial magnetofection reagent) was obtained from Beijing Chief-East Tech Co., Ltd. (Qwbio) (Beijing, China). 1-Ethyl-3-[3-(dimethylamino)propyl] carbodiimide (EDC) and *N*-hydroxy succinimide (NHS) were purchased from Chengdu Xiya Reagent Co. (Sichuan, China). PolyMag-200 and 96-well cell culturing magnetic plates were purchased from Chemicell GmbH (Berlin, Germany). Dulbecco’s modified Eagle’s medium (DMEM) and penicillin-streptomycin and fetal bovine serum (FBS) were obtained from Invitrogen-Gibco (CA, USA). Rhodamine B isothiocyanate was obtained from Aladdin Ind., Co. (Shanghai, China). LysoTracker Green DND-26 was obtained from Shanghai Wei Jin Biological Technology Co., Ltd. (Shanghai, China). The CCK-8 test kit was obtained from Seven Seas Biological Technology Co., Ltd. (Shanghai, China), and the Endo-free Plasmid Maxi Kit-25 was purchased from OMEGA (GA, USA). Phosphate-buffered saline (PBS) and other reagents were prepared in our laboratory. All the solvents and chemicals were of analytical grade.

### Synthesis of PEI-SPIO-NPs

PEI-SPIO-NPs were prepared as described previously [24]. Briefly, 0.1 g EDC and 0.5 g NHS were added to 15 mL carboxyl-modified SPIO-NPs aqueous solution (5 mg/mL, pH adjusted to 5), and the solution was stirred for 4–6 h at room temperature to activate the carboxyl of iron oxide nanoparticles. Next, the same volume of polyethylenimine hydrochloride aqueous solution (20 mg/mL) was added to react for several hours. The resulting conjugated PEI-SPIO-NPs solution was dialyzed using a dialysis membrane (MWCO 20,000) submerged in distilled water for 2 days to remove any unconjugated PEI molecules and intermediates. An aliquot of the nanoparticle solution was freeze-dried and stored.

### Synthesis and Characterization of PEI-SPIO-NPs/pDNA Complexes

The synthesis of PEI-SPIO-NPs was as described above, PEI-SPIO-NPs and plasmid DNA (pDNA) were pre-heated separately for 10 min at 37 °C, and then mixed together at different N/P ratios to prepare the PEI-SPIO-NPs/pDNA complexes. The morphology of the PEI-SPIO-NPs/pDNA complexes was measured by transmission electron microscopy (TEM, CM100, Philips, Netherlands), and the hydrodynamic diameter was measured by dynamic light scattering (DLS, Nicomp 380, PSS, FL, USA). Their magnetic properties of SPION and PEI-SPIO-NPs were examined with an EV-11 vibrating sample magnetometer (PPMS-9, Quantum Design, San Carlos, CA, USA) at 300 K. The measurements were normalized to the weight of iron in each sample, and the measurements were verified through inductively coupled plasma optical emission spectrometer (ICP-OES).

Surface charges were measured by determining the zeta potential using the dynamic light scattering instrument (Malvern, Southborough, MA, USA) in phosphate-buffered saline (PBS, pH = 7.4). The PEI-SPIO-NPs/pDNA complexes were prepared at various N/P mole ratios ranging from 2.5 to 25 by adding the PEI-SPIO-NPs solution to the pDNA solution, and the final pDNA concentration was adjusted to 30 μg/mL.

The components of the PEI-SPIO-NPs/pDNA complexes were detected using an atomic force microscope (IPC-208B, Chongqing University, Chongqing, China). A small, flaky metal that was used as a carrier for observation was washed with acetone and dried. Then, a few drops of the antisense probe sample were placed on the metal and air-dried. The surface morphology of the sample was observed in a large-scale scanning area of 700 nm × 700 nm and 1000 × 1000 pixels. The molecular structure and microstructure of the sample were observed in a small-scale scanning area of 9 nm × 9 nm and 800 × 800 pixels. The original image data were transmitted to a computer, and 3D reconstruction was performed with G2DR software [25]. Measurements were repeated thrice for each group.

The mobility of pDNA after binding with PEI-SPIO-NPs was analyzed by gel electrophoresis. The N/P ratios of PEI-SPIO-NPs/pDNA were 1/5, 1, 5, 10, 15, 20, 25, and 30, where the content of DNA was kept at 3 μg. After 15-min incubation at 37 °C, 10 μL of mixture solution was analyzed by 1% agarose gel electrophoresis (90 V, 30 min). Naked pDNA was used as a control.

The effects of PEI-SPIO-NPs on the protection of pDNA against DNase I were evaluated. PEI-SPIO-NPs/pDNA complexes at various N/P ratios and naked pDNA (3 μg) were incubated at 37 °C in a 5% CO_2_ sub-humid environment for 30 min with DNase I (4 U) in DNase/Mg^2+^ digestion buffer consisting of 50 mM Tris-HCl (pH= 7.6) and 10 mM MgCl_2_. DNase I was then inactivated by adding EDTA solution (pH= 8.0) until the final concentration was 2.5 mM. The sample was then incubated for 15 min at 65 °C, and 10 μL of 1 mg/mL sodium heparin was added to release the pDNA from PEI-SPIO-NPs/pDNA complexes [26]. The integrity of pDNA was assessed by 1% agarose gel electrophoresis (90 V, 30 min).

### Magnetic Field

The novel magnetic field generator (Fig. 4) was developed by our group in collaboration with the Electrical Engineering College of Chongqing University [20, 21]. We used the 3D CAD image to show the magnet arrangement of the uniform magnetic field. The XOY plane distribution and 3D distribution of the magnetic field in the region of interest were displayed. The distribution of PEI-SPION-NPs in different magnetic fields was observed, and a non-uniform magnetic field (96-well Nd-Fe-B permanent plate) was used as a control.

### Cell Culture

Human osteosarcoma cell line MG-63 (originally from American Type Culture Collection) was obtained from Chongqing Engineering Research Center of Stem Cell Therapy (Chongqing, China). The cells were maintained in DMEM supplemented with 10% FBS, 100 U/mL penicillin, and 100 mg/mL streptomycin and incubated at 37 °C with 5% CO_2_ and 95% relative humidity.

### In Vitro Cytotoxicity

The in vitro cytotoxic effects of different nanoparticles with or without pDNA and the magnetized or unmagnetized nanoparticles on the MG-63 osteosarcoma cells were determined using the CCK-8 test kit. The cells were seeded in 96-well culture plates at a density of 4 × 10^3^ cells per well, and different nanoparticles were added and incubated for 24, 48, 72, and 96 h. CCK-8 solution (10% volume of the culture medium) was added to each well at preset time points, and cells were cultured for another 2 h. The optical density of the cells was assessed using a fluorescence microplate reader at a wavelength of 450 nm, with the absorbance value reflecting the high cell viability; the observation of high viability indicates that nanoparticles induce low cytotoxic effects. PolyMag-200/pDNA and PolyMag-200 were used as positive control. To study the toxicity effects of different magnetic fields on cells, we selected PEI-SPIO-NPs(without pDNA) as gene vectors, and the optical density of cells intervened with different magnetic fields at different time points was investigated.

### Confocal Microscopy Analysis

The uptake of PEI-SPIO-NPs/pDNA complexes or polyethylenimine nanoparticles (PEI-NPs) was observed by confocal laser scanning microscopy. MG-63 osteosarcoma cells were seeded in 24-mm glass dishes at a density of 3 × 10^5^ per well. Rhodamine B isothiocyanate (RBITC)-labeled PEI-SPIO-NPs/pDNA (3 μg) complexes were added to the cells and incubated for 30 min under the action of uniform magnetic field or non-uniform magnetic field; RBITC-labeled PEI-NPs/pDNA complexes were added to the cells without the intervention of a magnetic field. The cells were then immediately washed twice with PBS (0.01 M) to remove any free RBITC-labeled PEI-SPIO-NPs/pDNA complexes or RBITC-labeled PEI-NPs/pDNA complexes, and the treated cells were incubated for 6–24 h. The medium was removed 6, 12, and 24 h post-transfection, and cell nuclei were stained with Hoechst 33342 for 5  min. After removal of the Hoechst 33342 residue, the cells were incubated in pre-warmed medium containing 0.5 mM LysoTracker Green DND-26 for 1 h and then washed twice with pre-warmed PBS. The cells were imaged under a confocal laser scanning microscopy (LSM 700, Carl Zeiss, Oberkochen, Germany) with a × 40 objective to visualize the fluorochromes with the following excitation (Ex) and emission (Em) wavelengths [GFP (Ex: 488 nm; Em: 530 nm), Hoechst (Ex: 350 nm; Em: 460 nm), LysoTracker Green (Ex: 443 nm; Em: 505 nm), and RBITC (Ex: 554 nm; Em: 576 nm)] [27].

### Flow Cytometry Analysis

MG-63 cells were seeded in 12-well plates (1 × 10^5^ cells per well) for 24 h, after which they were rinsed with PBS twice and pre-incubated for 1 h with 0.8 mL Opti-MEM medium at 37 °C prior to transfection. The PEI-SPIO-NPs/pDNA complexes (N/P = 10) or PEI-NPs/pDNA complexes (N/P = 10) containing 3 μg pDNA were added to each well, and the cell culture plates were placed in the uniform or non-uniform magnetic field for 20 min. PolyMag-200/pDNA was used as positive controls. After 4 h, the cells were rinsed three times with 1 mL PBS (0.01 M) to remove any free PEI-SPIO-NPs/pDNA complexes or PEI-NPs/pDNA complexes, and the cells were cultured for another 24 h. After incubation, the cells were collected and evaluated for transfection efficiency by flow cytometry (BD FACS Canto II, BD Biosciences, San Jose, CA, USA), this part of the experiment was repeated three times.

### Statistical Analysis

Quantitative data were obtained in triplicate (*n* = 5) and expressed as the mean ± standard deviation. Statistical analyses were performed using one-way ANOVA for multiple comparisons and the Student’s *t* test for intergroup comparison. A *P* value < 0.05 was considered statistically significant.

## Results and Discussion

### Principle of Magnetofection

**Fig. 1:**
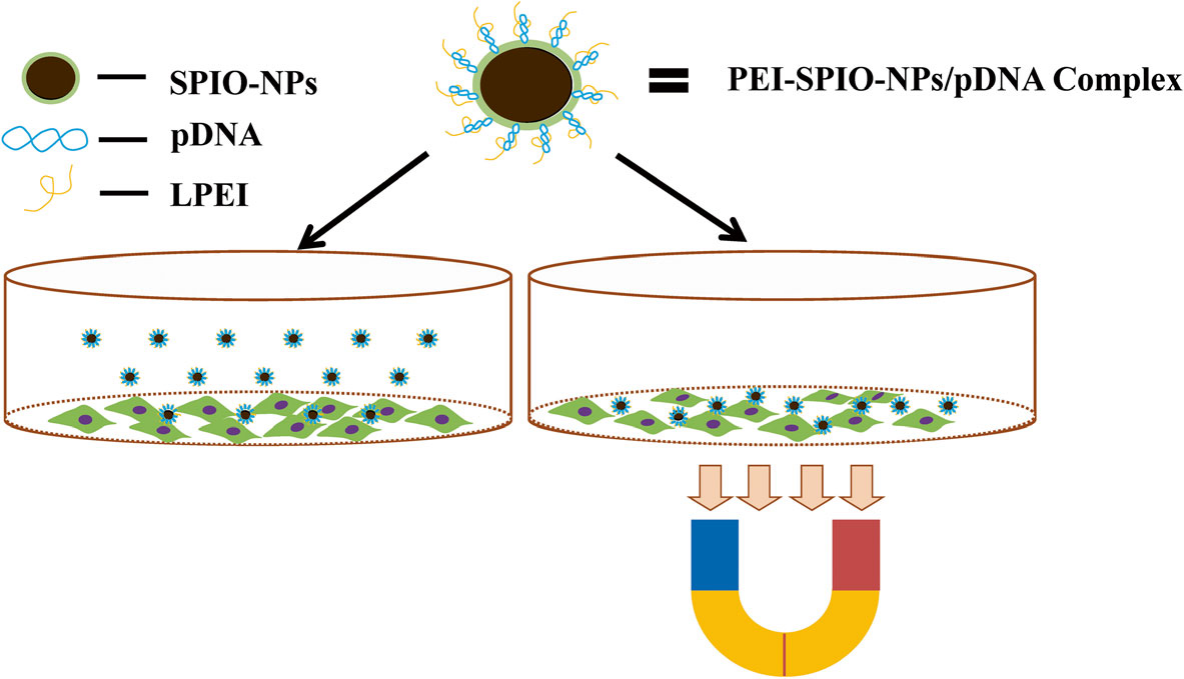
Overview of magnetofection using PEI-SPIO-NPs/pDNA complex. Lined polyethyleneimine (LPEI) and superparamagnetic iron oxide nanoparticles (SPIO-NPs) are associated by the reaction of dehydration condensation. For this purpose, SPIO-NPs were coated with LPEI, i.e., the LPEI is tightly bound to the surface of SPIO-NPs and they form PEI-SPIO-NPs together. If PEI-SPIO-NPs are mixed with naked pDNA, negatively charged pDNA binds to positively charged PEI-SPIO-NPs via electrostatic adsorption. Cells are incubated with the PEI-SPIO-NPs/pDNA complexes in the presence of a magnetic field which attracts the complexes toward the cells. The result of magnetofection is that essentially all cells come into contact with vectors and a high percentage of cells are rapidly transfected

Magnetofection is defined as vectors that are associated with superparamagnetic nanoparticles and that accumulate on the target cells by the application of magnetic gradient fields [15]. Figure 1 presents the components and morphology of PEI-SPIO-NPs/pDNA complexes and the primary principle of magnetofection. Slow vector accumulation and consequently low vector concentration in target tissues have been identified as simple but strong barriers to effective gene delivery [28]. The major efficacy-enhancing mechanism of magnetofection appears to be the rapid sedimentation of the full vector dose on the target cells, such that up to 100% of these cells will have vector particles bound to their surfaces within a few minutes. Thus, magnetofection is an appropriate tool with which to overcome the strong barrier of slow vector accumulation and, consequently, low vector concentration at target tissues. PEI-SPIO-NPs/pDNA complexes randomly, and is limitedly and time-consuming in its deposition in the targeted cells in the absence of a magnetic field. Nevertheless, PEI-SPIO-NPs/pDNA complex can broadly and uniformly touch the targeted cells in a relatively short period of time in the presence of a magnetic field, which may increase the chance of endocytic uptake in unit area of cells, so as to improve the utilization rate of PEI-SPIO-NPs/pDNA complex and enhance the transfection efficiency.

### Characterization of PEI-SPIO-NPs/pDNA Complexes

**Fig. 2:**
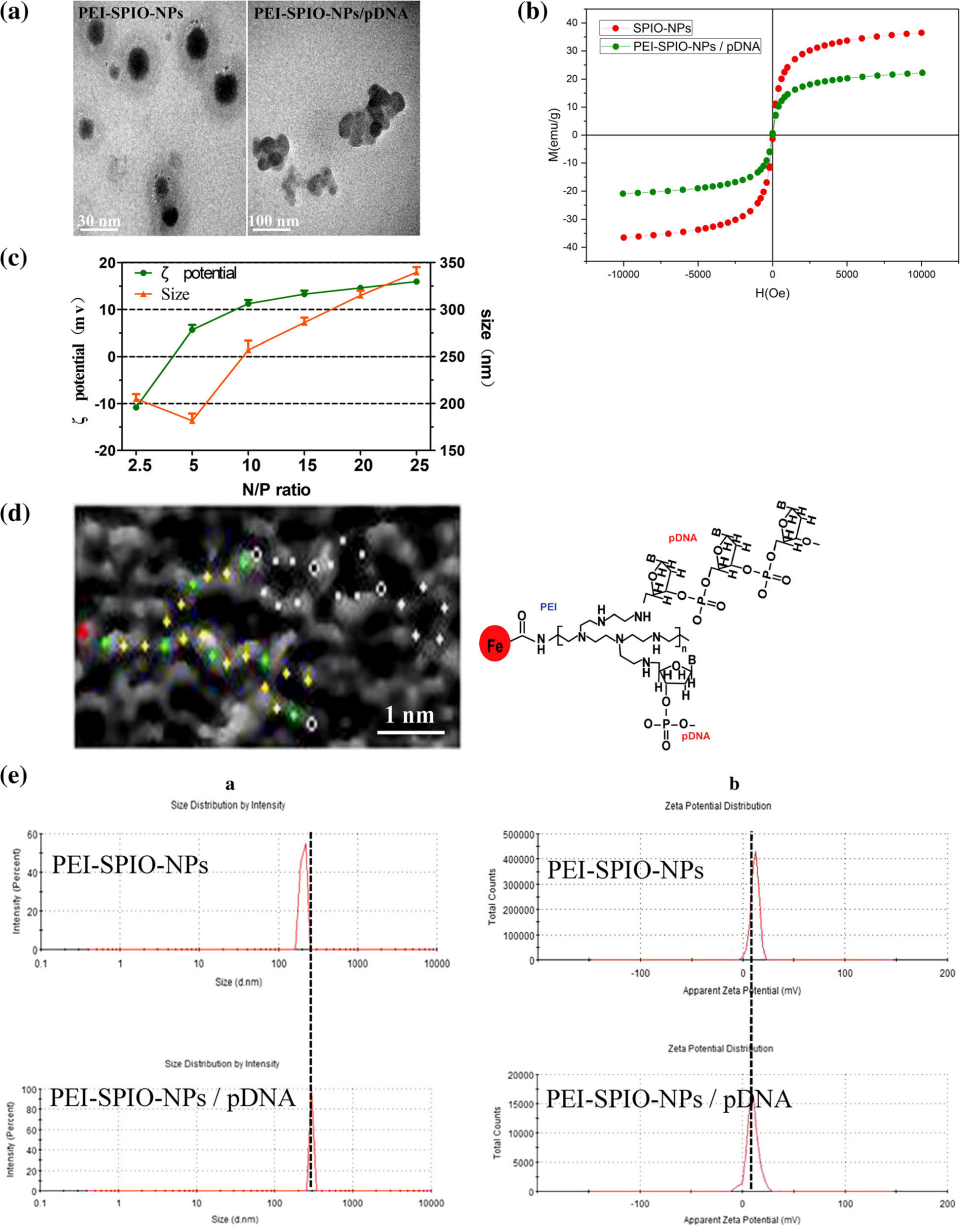
Characteristics of PEI-SPIO-NPs/pDNA complexes. **a** TEM of PEI-SPIO-NPs and PEI-SPIO-NPs/pDNA complexes. **b** Hysteresis loops of SPIO-NPs and PEI-SPIO-NPs/pDNA complexes. **c** Zeta potential and hydrodynamic diameter of PEI-SPIO-NPs/pDNA complexes at various N/P ratios. **d** AFM grayscale image and the molecular structure of the PEI-SPIO-NPs/pDNA complexes. Red dots represent the location of the SPIO chemical group, the green dots indicate nitrogen atoms, yellow dots signify carbon atoms and the phosphorus atom is represented by a white circle black dot. **e** (a) Size distribution of PEI-SPIO-NPs/pDNA complexes and **e** (b) zeta potential of PEI-SPIO-NPs/pDNA complexes as measured by a Malvern Zetasizer dynamic light scattering instrument. Results are expressed as the mean ± SD (*n* = 5)

Figure 2a shows that the PEI-SPIO-NPs are roughly spherical and PEI-SPIO-NPs/pDNA complexes appear aggregated, both having favorable dispersibility, and the attraction between positive and negative charges renders PEI-SPIO-NPs/pDNA complexes able to acquire a small size. The hysteresis loop of PEI-SPIO-NPs/pDNA complexes is shown in Fig. 2b. The weight percent of iron oxide in PEI-SPION-NPs measured by an atomic absorption spectrometer is 20.33(± 2.87) %. The saturation magnetization of the PEI-SPIO-NPs/pDNA complexes was 21.5 (± 1.6) emu/g of iron. Despite the reduced magnetic properties compared to that of the unmodified superparamagnetic iron oxide nanoparticles, PEI-SPIO-NPs/pDNA complexes exhibited excellent magnetic responsiveness, which is necessary for high efficiency magnetofection.

Hydrodynamic diameter and zeta potential were regarded as the indispensable parameters of gene carriers. Figure 2c shows that the hydrodynamic diameter of PEI-SPIO-NPs/pDNA complexes was 200 (± 21.7) nm with an N/P ratio (NH_2_—group in PEI / PO_4_—group in pDNA) of 2.5, and 175 (± 16.4) nm when N/P ratio was 5, indicating that the rapid change in size occurred during transition of the potential from negative to positive. Thereafter, the repulsive forces between the PEI-SPIO-NPs/pDNA complexes increased with higher N/P ratios, indicating that the size of nanoparticles had increased.

We analyzed the types of chemical bonds according to the arrangement of atoms, and further speculated on the molecular structure of PEI-SPIO-NPs/pDNA complexes. The molecular structure of PEI-SPIO-NPs/pDNA complexes was indirectly disclosed by atomic force microscopy (AFM, Fig. 2d), which showed that the carboxyl groups of superparamagnetic iron oxide nanoparticles combined with the primary amine of PEI by the amide bond. In addition, the pDNA was wrapped in a PEI molecular chain via electrostatic binding between the phosphate groups of the nucleotide chain and the amine groups of PEI, forming PEI-SPIO-NPs/pDNA complexes. In the AFM gray-scale image, the red dot represents the location of the iron atoms, the green dot indicates nitrogen atoms, the yellow dots signify carbon atoms, and the phosphorus atom is represented by a white circle with a black dot.

As shown in Fig. 2e, the potential of PEI-SPIO-NPs/pDNA complexes was detected using the dynamic light scattering instrument. At pH = 7, the potential of the superparamagnetic iron oxide nanoparticles (SPIO-NPs) was − 6.6 (± 1.1) mV, which indicated the presence of carboxylic groups at their surface. The potential of PEI-SPIO-NPs was + 18.2 (± 1.5) mV. The potential of PEI modified with SPIO-NPs was transformed into a positively charged surface, which could be used as a gene carrier that combine with negatively charged pDNA. We evaluated the change in surface charges of PEI-SPIO-NPs/pDNA complexes at different N/P ratios. The PEI-SPIO-NPs/pDNA complexes showed a negative surface charge even at a low N/P ratio of 2.5, which gradually translated into a positive surface charge as the N/P ratio increased to 5. This increase in N/P ratios in PEI-SPIO-NPs/pDNA complexes resulted in an increase in the positive surface charge of polyplexes. At an N/P ratio of 10, the potential of PEI-SPIO-NPs/pDNA complexes (N/P = 10) was + 11.9 (± 1.2) mV, and the surface charge reached a plateau. The positively charged complexes came in contact with the negatively charged cell membrane and enabled cellular uptake of the complexed nanoparticles [29]. PEI-SPIO-NPs can not only be used as gene carriers of negatively charged pDNA but also contribute a certain level of magnetism for targeted drug delivery, and become the contrast agent used for magnetic resonance imaging (MRI) contrast imaging [30, 31].

**Fig. 3:**
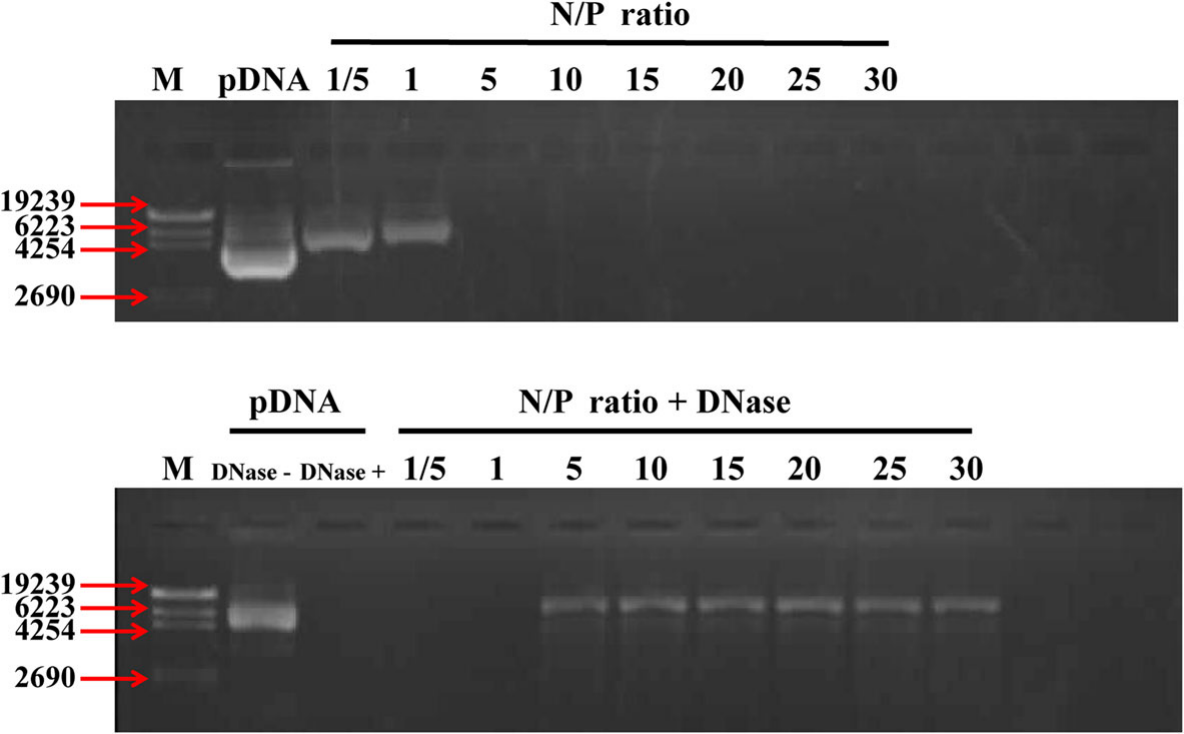
The pDNA binding assay and the pDNA protection assay of PEI-SPIO-NPs. **a** Agarose gel electrophoresis of PEI-SPIO-NPs/pDNA complexes at various N/P ratios. **b** Electrophoretic mobility analysis of PEI-SPIO-NPs/pDNA after DNase-I treatment. PEI-SPIO-NPs at various N/P ratios and pDNA (3 μg) were incubated at 37 °C in a 5% CO_2_ sub-humid environment for 30 min with DNase-I (4 U) in DNase/Mg^2+^ digestion buffer consisting of 50 mM Tris-HCl (pH = 7.6) and 10 mM MgCl_2_. DNase I was then inactivated by adding EDTA solution (pH = 8) until the final concentration was 2.5 mM. The sample was then incubated for 15 min at 65 °C, and 10 μL of 1 mg/mL sodium heparin was added to release pDNA from PEI-SPIO-NPs/pDNA. The integrity of pDNA was assessed by 1% agarose gel electrophoresis (90 V, 30 min)

A fundamental requirement of gene carriers is that the transfection carrier must efficiently form a stable complex with nucleic acids. To evaluate its binding ability, similar volumes of PEI-SPIO-NPs solution and pDNA solution were mixed at different N/P ratios and vortexed. A 10-μL aliquot of the mixture was then analyzed by agarose gel electrophoresis (Fig. 3a). In contrast to naked pDNA, the migration of the plasmid was completely blocked at an N/P ratio of 5, indicating that the PEI-SPIO-NPs fully concentrated the pDNA [32].

Another feature of PEI-SPIO-NPs as potential gene carriers is that they could protect pDNA from degrading by nucleases, thereby favoring transfection. Figure 3b shows that naked pDNA is significantly degraded by DNase-I. The PEI-SPIO-NPs/pDNA complexes at a N/P mole ratio < 5 were totally digested, whereas pDNA released from PEI-SPIO-NPs/pDNA complexes (N/P = 5:1) remained intact. The results of these DNase-I protection assays showed that the PEI-SPIO-NPs effectively protected pDNA from DNase-I digestion, thus implying its potential applications to gene therapy.

As shown above, when N/P was < 5, PEI-SPIO-NPs could not protect pDNAs from nuclease degradation. The sizes of PEI-SPIO-NPs/pDNA complexes increased when N/P was > 10, which was unsuitable for vector transport [33, 34]. The size of the PEI-SPIO-NPs/pDNA complexes was the smallest and exhibited stability at N/P = 5. Moreover, the zeta potential of the PEI-SPIO-NPs did not significantly increase when N/P > 10. Therefore, to ensure the stability of PEI-SPIO-NPs/pDNA, an N/P ratio of 10 was selected for the subsequent experiments.

### Generation of a Magnetic Field

**Fig. 4:**
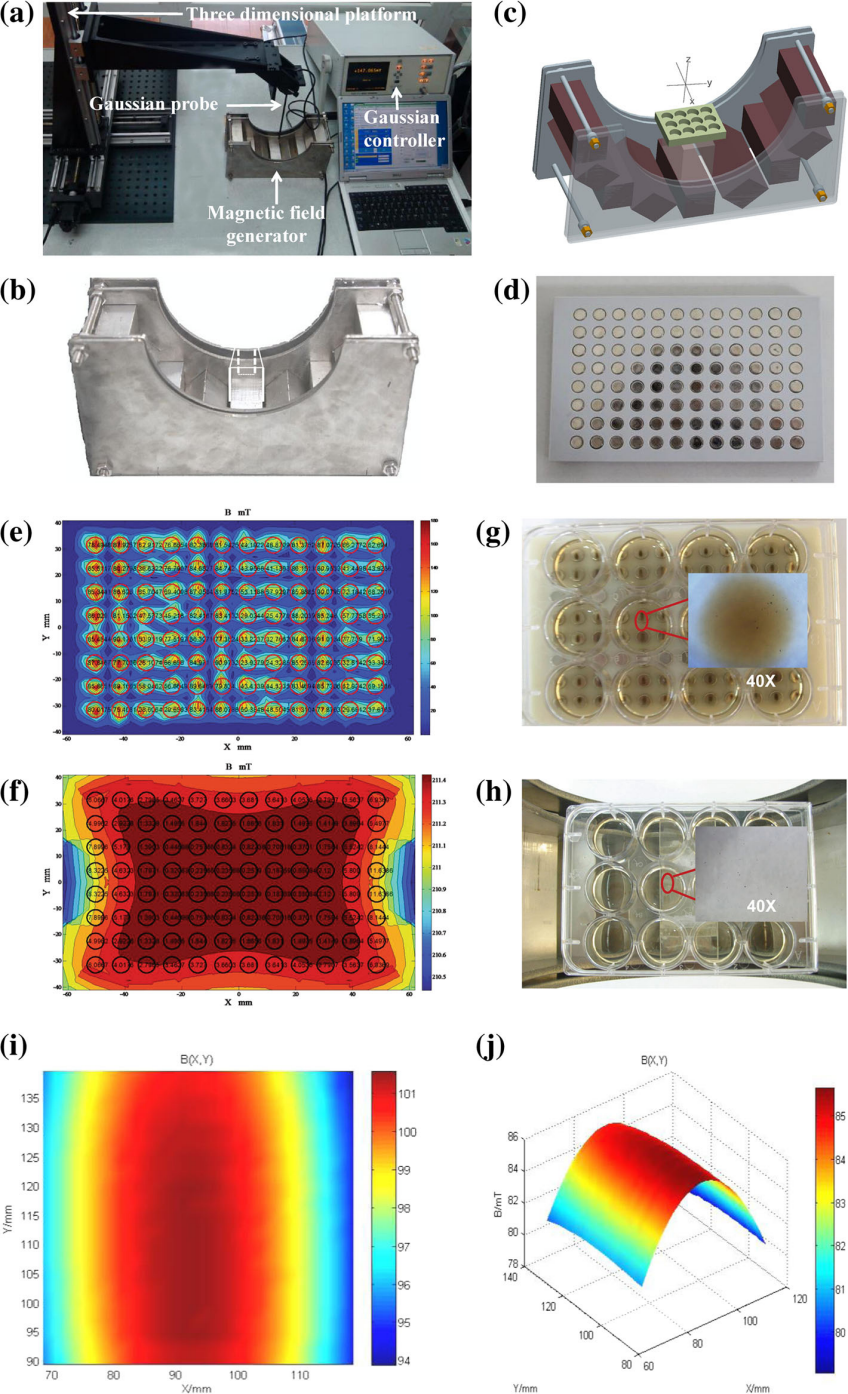
The magnetic field generator and their magnetic field uniformity and PEI-SPIO-NPs distributed in the different magnetic field. **a** Measurement of magnetic field uniformity by Graussmeter. **b** Uniform magnetic field generator. **c** 3D image of magnet arrangement of uniform magnetic field generator (where each magnet has a size of 40 × 40 × 200 mm^3^). **d** 96-well magnetic field generator. **e** Magnetic field uniformity of 96-well magnetic field generator. **f** Magnetic field uniformity of uniform magnetic field generator. **g** Distribution of PEI-SPIO-NPs in 96-well magnetic field. **h** Distribution of PEI-SPIO-NPs in uniform magnetic field. **i** The XOY plane distribution of uniform magnetic field (50 mm × 50 mm) and **j** 3D distribution of uniform magnetic field

The novel Halbach magnetic field generator (Fig. 4a, b) was developed by our group, in collaboration with the Electrical Engineering College of Chongqing University [20, 21]. The novel magnetic field generator is composed of nine identical cuboid permanent magnet modules and two passive shimming sheets (Fig. 4c), which generate a highly uniform magnetic field in the horizontal plane.

The optimizing magnet structure can generate a magnetic field that flatly distributes in the horizontal direction of the 50 mm × 50 mm area in the YOZ plane. The gradient is distributed in the vertical direction with a gradient of 2 mT/mm. The magnetic field is uniformly distributed in an XOY area of 50 mm × 50 mm with uniformity of 1.3 × 10^−3^ and a magnetic field of 0.0739 T, the magnetic strengths of coronal plane and sagittal planes where the cell-culturing plate was placed are respectively 0.0632 T and 0.07 T. The uniformity difference of each well in the 96-well cell culturing magnetic plate was approximately 80% (Fig. 4d, e). However, the uniformity difference of each well in the novel Halbach magnetic field is smaller than 2‰, so the uniformity difference between the two magnetic fields is > 100-fold (Fig. 4f). In this case, we set a 96-well cell culturing magnetic plate as a non-uniform magnetic field, and the novel Halbach magnetic field is a relatively uniform magnetic field, which was used as an experimental tool for subsequent studies.

The distribution of PEI-SPIO-NPs was significantly affected by the magnetic field. PEI-SPIO-NPs sank gradually due to gravity and were randomly distributed in the absence of the magnetic field. Upon the application of a magnetic field, PEI-SPIO-NPs rapidly sank to the bottom of the plates. Furthermore, the distribution could also be significantly changed in different magnetic fields. In the conventional non-uniform magnetic field (96-well cell culturing magnetic plate), PEI-SPIO-NPs were gathered into masses or bands and distributed along with the lines of magnetic force (Fig. 4g). However, the PEI-SPIO-NPs were uniformly distributed in the uniform magnetic field that was induced by the novel magnetic field generator (Fig. 4h). As can be seen from the XOY plane distribution and 3D distribution of the magnetic field (Fig. 4i, j), the red area in the figure is a relatively uniform magnetic field, with the uniformity of about two thousandths (*Z* = 10 mm), and it can be seen that the gradient of the magnetic field in the target region is about 1.3 t/m (130 G/cm). The magnetic field generated in the designated area could obtain a good flat property in the horizontal direction by adjusting the arrangement of the magnet module and changing the magnetization direction of the magnet module.

### Assessment of In Vitro Cytotoxicity

**Fig. 5:**
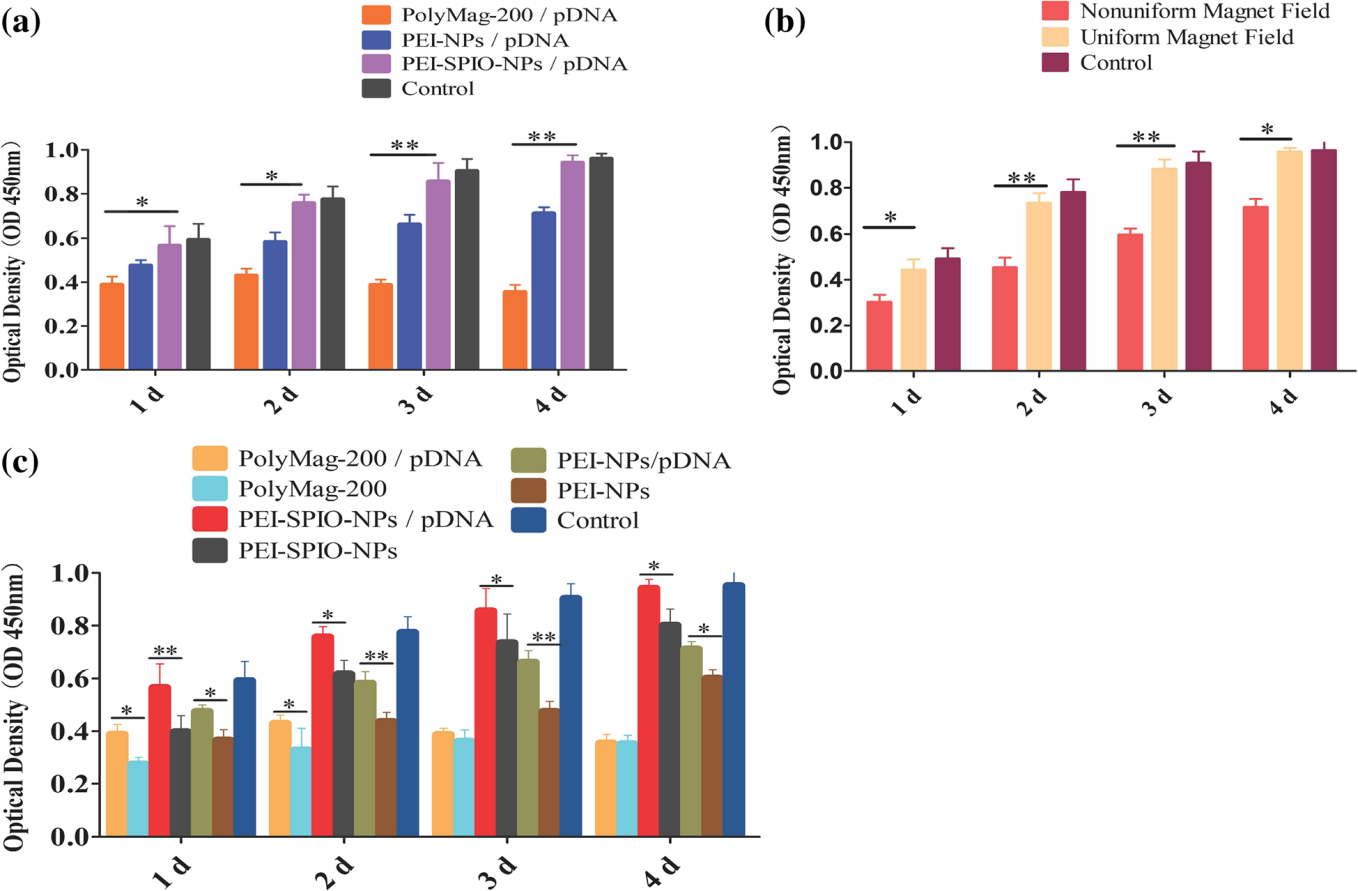
Comparison of cytotoxicity effects of the uniform magnetic field and non-uniform magnetic field, PEI-SPIO-NPs/pDNA complexes, PolyMag-200/pDNA complexes and PEI-NPs/pDNA complexes, and different nanoparticles with pDNA and without pDNA on MG-63 osteoblasts. Cytotoxicity is detected by the CCK-8 test kit, the absorbance was measured at a wavelength of 450 nm, which reflects the cell viability, and the high viability is indicative of the low cytotoxicity. The results are expressed as the mean ± SD (*n* = 5, one-way ANOVA, *P < 0.05, **P < 0.01). **a** The cytotoxicity effects of PEI-SPIO-NPs/pDNA complexes, PolyMag-200/pDNA complexes and PEI-NPs/pDNA complexes on MG-63 osteoblasts. **b** The cytotoxicity effects of the uniform magnetic field and non-uniform magnetic field on MG-63 osteoblasts and **c** the cytotoxicity effects of different nanoparticles with pDNA and without pDNA

We used CCK-8 kits to evaluate the effects of magnetization, magnetic field, and pDNA on the cytotoxicity of nanoparticles. Figure 5a shows that with the prolongation of culture time, the absorbance values increased in all the groups except for the PolyMag-200/pDNA groups. The absorbance values of PEI-SPIO-NPs/pDNA group were higher than that of PEI-NPs/pDNA group and PolyMag-200/pDNA group (*P* < 0.05) and lower than that of the control group (*P* > 0.05), suggesting that the cytotoxicity of PEI-SPIO-NPs/pDNA complexes is lower than unmagnetized PEI-NPs/pDNA complexes and PolyMag-200/pDNA complexes. Figure 5b shows the negative effects on the absorbance values caused by the uniform and non-uniform magnetic fields. The negative effect caused by the uniform magnetic field was smaller than that caused by the non-uniform magnetic field (*P* < 0.05). As shown in Fig. 5c, the absorbance values of nanoparticles without pDNA is significantly lower than that of nanoparticles with pDNA (*P* < 0.05), which meant nanoparticles added with pDNA have low cytotoxicity effect to Mg-63 osteoblasts.

Although PEI is an efficient transfection reagent, its cytotoxicity is strongly and positively correlated with its transfection efficiency and the mechanism of cytotoxicity caused by PEI is not very clear [35]. The present study found that during transfection, PEI increases the permeability of the cell membrane and damages the integrity of the mitochondrial and nuclear membranes [36, 37]. Sonawane et al. [38] reported that PEI25K promotes the release of mitochondrial protons and inhibits the electron transport chain in a dose- and time-dependent manner, indicating that PEI induces cell apoptosis. Another study showed that PEI induces cell autophagy, which is closely related to cytotoxicity [39]. The cytotoxicity of magnetized PEI decreased, and this might be attributable to the surface carboxyl groups of superparamagnetic iron oxide nanoparticles, which are negatively charged, thus partly neutralizing the positive charge of the PEIs and decreasing the chances of incurring irreversible damage. The non-uniform magnetic field induced PEI-SPIO-NPs/pDNA complexes to gather into masses or bands, and be distributed along with the lines of magnetic force (Fig. 4b), thereby causing severe damage to parts of the cell membrane and even cell death due to the excessively high number of positive charges [40]. In contrast, PEI-SPIO-NPs/pDNA complexes were uniformly distributed across the uniform magnetic field, thereby decreasing the accumulation of positive charges and reducing the cytotoxicity.

Nanoparticles added with pDNA have low cytotoxicity effect to Mg-63 osteoblasts than nanoparticles without pDNA; this may be attributed to the negatively charged pDNA partially neutralize the surface positive potential of the cationic nanoparticles at the beginning of magnetofection progress. Moghimi SM et al. concluded that PEI-induced cellular toxicity could been defined as a two-stage process, with the first stage taking place within 30 min of PEI uptake [41]. Stage-one toxicity has been defined as necrosis that is based on compromise of cell membrane integrity mediated by PEI binding to negatively charged plasma membrane proteoglycans, with highly cationic NPs being extremely cytotoxic [42]. After internalized by MG-63 osteoblast, different nanoparticles exhibits similar cytotoxic mechanisms, internalization leads to proton buffering, osmotic pressure, and eventual lysis of lysosomal membranes, releasing hydrolytic enzymes and other lysosomal constituents into the cytoplasm [43].

### Cellular Uptake of PEI-SPIO-NPs/pDNA Complexes

To better understand the intracellular distribution of PEI-SPIO-NPs/pDNA complexes or PEI-NPs/pDNA complexes and the relationship between intracellular uptake of complexes and transfection efficiency, a laser scanning confocal microscope was used to trace the magnetized RBITC-PEI-SPIO-NPs/pDNA complexes and non-magnetized RBITC-PEI-NPs/pDNA complexes during transfection of MG-63 cells. LysoTracker Green D26 is a specific marker for lysosomes, and Hoechst 33342 specifically stains nuclei. Overlapping green and red fluorescence, which yields yellow fluorescence, represents colocalization and indicates entrapment of polyplexes in lysosome.

**Fig. 6:**
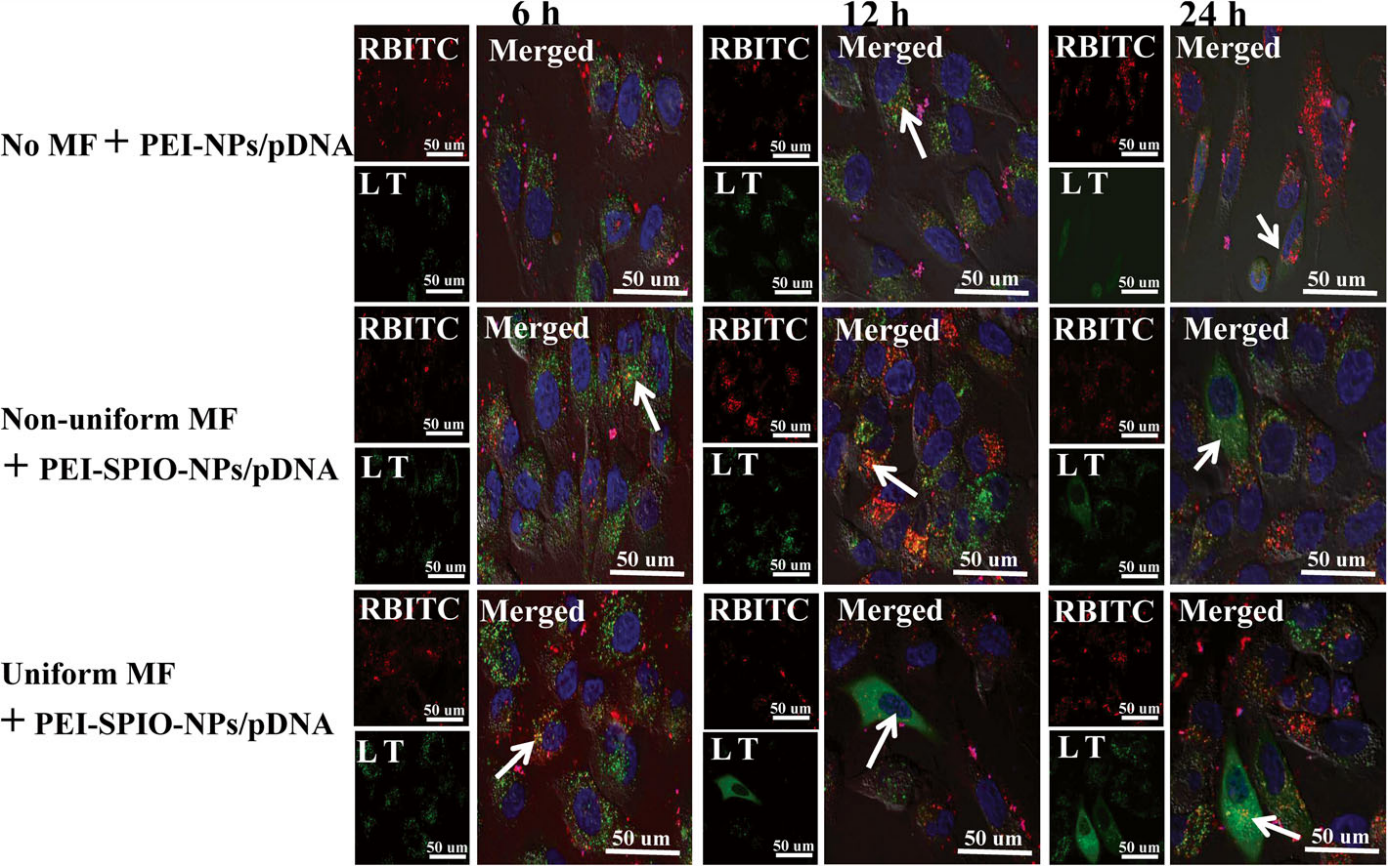
Intracellular tracking of PEI-SPIO-NPs/pDNA complexes in the presence of uniform magnetic field (uniform MF) or non-uniform magnetic field (non-uniform MF) and PEI-NPs/pDNA complexes without magnetic field (No MF) at 6, 12, and 24 h post-transfection to MG-63 osteoblasts. Confocal images were obtained from three channels and overlaid: red indicates RBITC-PEI-SPIO-NPs/pDNA complexes (excitation: 554 nm; emission: 576 nm), green represents lysosomes stained with Lysotracker-DND26 and the homogeneous green in the cytoplasm implies GFP expression (excitation: 443 nm; emission: 505 nm), blue signifies the nuclei stained by the Hochest 33342. (excitation: 359 nm; emission: 461 nm)

Figure 6 shows that extensive co-localization was observed at 6 h post-transfection, which indicates that most of the complexes were entrapped in endosomes. The RBITC-PEI-SPIO-NPs/pDNA + uniform magnetic field group showed a higher frequency of co-localization than the RBITC-PEI-SPIO-NPs/pDNA + non-uniform magnetic field group and the RBITC-PEI-NPs/pDNA group (arrow). At 12 h post-transfection, most of the red fluorescence had already translocated from the green fluorescence of lysosomes to the surrounding blue fluorescence of nuclei, and some green fluorescence of gene expression was visible in the cytoplasm, which indicated that most complexes escaped the endosomes via the proton sponge effect [44, 45] (arrow). The fluorescent signals of the RBITC-PEI-SPIO-NPs/pDNA + uniform magnetic field group were more distinct than those of the RBITC-SPIO-PEI-NPs/pDNA + non-uniform magnetic field group and the RBITC-PEI-NPs/pDNA group. It was interesting that the cytoplasm emitted green fluorescence in the RBITC-PEI-SPIO-NPs/pDNA + uniform magnetic field group, which indicated expression of GFP, thereby illustrating the proton sponge effect that facilitates gene expression. At 12 h post-transfection, some nuclei emitted red fluorescence in the RBITC-PEI-SPIO-NPs/pDNA + uniform magnetic field group, indicating that complexes had been transported into nuclei, which may be attributable to the mechanical effect of magnetofection (arrow). At 24 h post-transfection, the green fluorescence in the cytoplasm showed maximal levels, the RBITC-PEI-SPIO-NPs/pDNA + uniform magnetic field group showed more intense green fluorescence than the RBITC-PEI-SPIO-NPs/pDNA + non-uniform magnetic field and the PBITC-PEI-NPs group (arrow). The affiliated magnetic field enabled the magnetic nanoparticles to attach rapidly to the cell membrane and accelerate intracellular uptake of magnetic nanoparticles. By contrast, up to 100% of these cells will have vector particles bound to their surfaces within a few minutes in the presence of the novel uniform magnetic field; more vectors adherence leads to a greater probability of cellular uptake, and transfection efficiency is positively correlated with uptake capability [46].

### In Vitro Transfection

Because PEI-SPIO-NPs are endowed with promising attributes such as pDNA condensation ability and high cell viability, we next used it as a gene carrier to determine the role of novel uniform and non-uniform magnetic fields on transfection efficiency. To evaluate the effectiveness of magnetofection, we selected the MG-63 osteosarcoma cell line as target cells and used GFP-pDNA as the reporter gene.

**Fig. 7:**
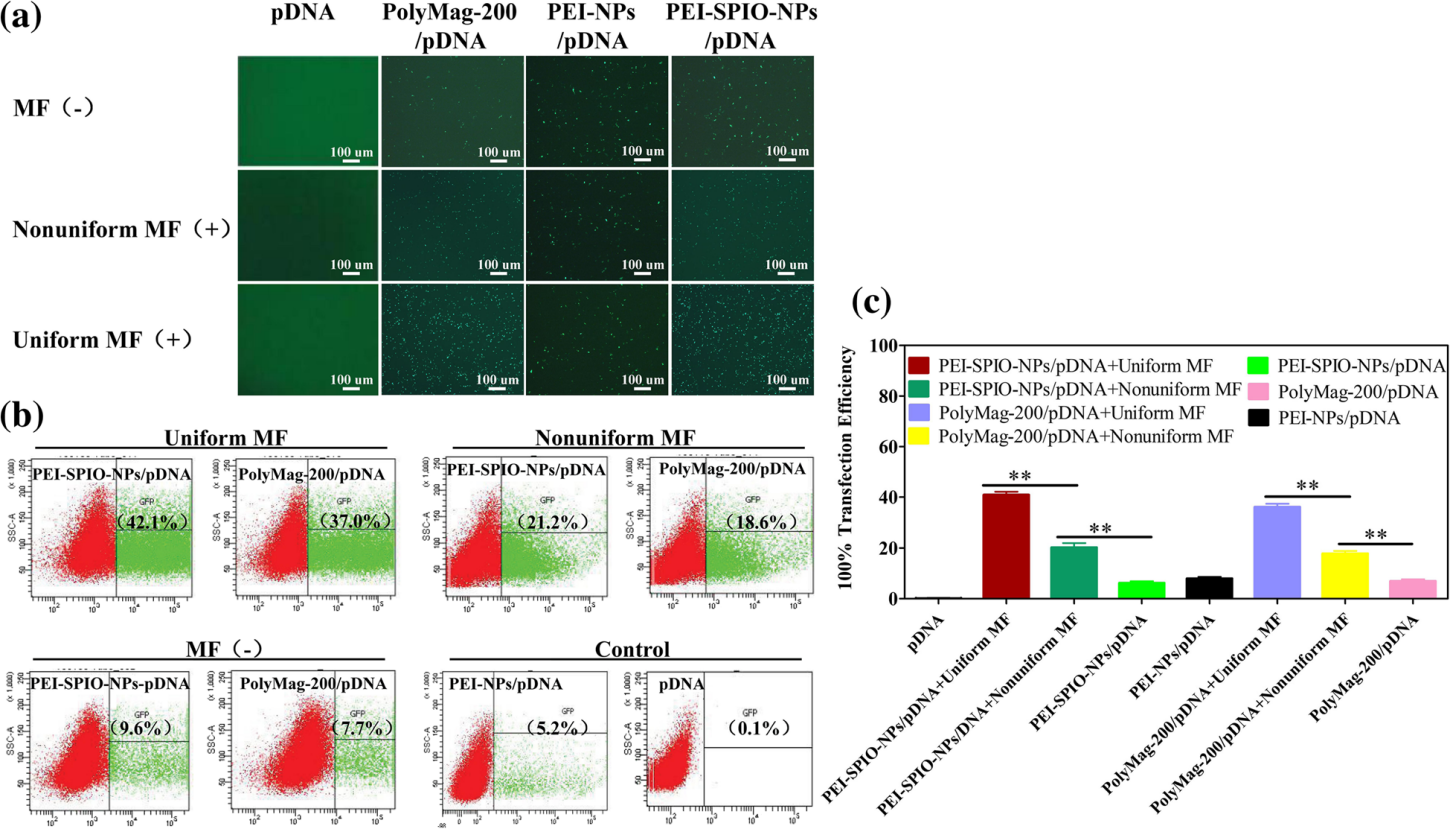
MG-63 osteoblasts transfected with PEI-SPIO-NPs/pDNA or PEI-NPs/pDNA under the condition of no magnetic field, non-uniform magnetic field, or uniform magnetic field. GFP-pDNA was used for reporter gene in combination with PEI-NPs or PEI-SPIO-NPs (N/*P* = 10), and the cells were exposed to the non-uniform or uniform magnetic fields for 20 min. At 24-h post-transfection, cell images were captured under an inverted fluorescent microscope. PolyMag-200 comprise commercial magnetic transfection reagents that were used as positive control, naked pDNA was used as the negative control. **a** At × 40 magnification in an inverted fluorescent microscope. **b** Transfection efficiency was calculated by flow cytometer and **c** statistical analysis of the transfection efficiency of PEI-SPIO-NPs/pDNA group, PolyMag-200/pDNA group and PEI-NPs/pDNA group. The results are expressed as the mean ± SD (*n* = 5, one-way ANOVA, *P < 0.05, **P < 0.01)

GFP expression was observed by inverted fluorescence microscopy at 24 h post-transfection. Figure 7a shows that the PEI-SPIO-NPs/pDNA group yielded significantly higher transfection efficiencies than the PEI-NPs/pDNA group in the presence of the uniform or non-uniform magnetic field, which is obvious in the uniform magnetic field group (*P* < 0.05), the transfection efficiency of the uniform magnetic field group was 42.1%, which is roughly two times higher than that of the non-uniform magnetic field group. Although PolyMag-200/pDNA showed high transfection efficiency and has been confirmed to transfect most adherent cell lines, it is also highly cytotoxic. The cells were collected for flow cytometry at 48 h post-transfection (Fig. 7b), and the statistical analysis results were in agreement with the findings from fluorescence microscopy (Fig. 7c).

The mechanism underlying the increase in transfection efficiency by magnetofection is currently unclear. Previous studies have demonstrated that magnetofection does not involve magnetic nanoparticles being pulled directly into the cells by the magnetic field, magnetic nanoparticles enter cells via endocytosis and remain intact upon cellular uptake [47]. The observed significant increase in transfection efficiency may be due to the synergistic effect of accelerated sedimentation and fast internalization [48, 49]. The number of magnetic complexes that enter each cell apparently influences pDNA content. Although genes must still escape endosomes before transcription, transfection efficiency is positively correlated with uptake capability [50], branched PEIs showed a higher uptake rate. However, once inside the cell, lysosomal escape becomes the key to transfection, especially with higher N/P ratios, and linear PEIs showed higher rates of lysosomal escape [51–53]. Because of the magnetic force, PEI-SPIO-NPs can quickly come in close contact with cell membranes, which to some extent, increases the uptake capability of complexes. Once the complexes are inside the cell, PEIs provide a structural advantage and facilitate timely release of pDNA, which is eventually expressed by the cells.

## Conclusions

The novel magnetic field generator has been developed to induced a uniform magnetic field, in which the PEI-SPIO-NPs/pDNA complexes are rapidly and uniformly distributed on the surface of MG-63 osteoblasts, thereby averting local transfection and decreasing disruption of the membrane caused by centralization of positively charged PEI-SPIO-NPs/pDNA complexes, ultimately resulting in an increase in the effective coverage of the magnetic gene carriers during transfection, and improving magnetofection efficiency. This innovative uniform magnetic field could be used to determine the optimal amount of PEI-SPIO-NPs and pDNA, and screen for the optimal formulation design of a magnetic gene carrier under homogeneous conditions. Most importantly, the novel uniform magnetic field facilitates the transfection of PEI-SPIO-NPs/pDNA complexes into osteoblasts, which provides a novel approach for the targeted delivery of therapeutic genes to osteosarcoma tissues and serves as a reference for the treatment of other tumors. However, a series of comprehensive studies are warranted to establish the therapeutic potential of PEI-SPIO-NPs that are integrated into a novel uniform magnetic field in combating osteosarcoma.

## Abbreviations

AFM: Atomic force microscopy
CLSM: Confocal laser scanning microscopy
DLS: Dynamic light scattering
DMEM: Dulbecco’s modified Eagle’s medium
EDC: 1-Ethyl-3-[3-(dimethylamino) propyl] carbodiimide
FBS: Fetal bovine serum
GFP: Green fluorescent protein
ICP-OES: Inductively coupled plasma optical emission spectrometer
MF: Magnetic field
MG-63: Human osteoblasts
MRI: Magnetic resonance imaging
MWCO: Molecular weight cut off
NHS: *N*-hydroxy succinimide
PBS: Phosphate-buffered saline
pDNA: Plasmid DNA
PEI: Polyethylenimine
PEI-NPs: Polyethylenimine nanoparticles
PEI-SPIO-NPs: Polyethylenimine modified superparamagnetic iron oxide nanoparticles
RBITC: Rhodamine B isothiocyanate
SPIO-NPs: Superparamagnetic iron oxide nanoparticles
TEM: Transmission electron microscopy
VSM: Vibrating sample magnetometer

## References

[1] Friebele JC, Peck J, Pan X, Abdel-Rasoul M, Mayerson JL (2015). Osteosarcoma: a meta-analysis and review of the literature. Am J Orthop (Belle Mead NJ).

[2] Misaghi A, Goldin A, Awad M, Kulidjian AA (2018). Osteosarcoma: a comprehensive review. SICOT J.

[3] Wedekind MF, Wagner LM, Cripe TP (2018). Immunotherapy for osteosarcoma: where do we go from here?. Pediatr Blood Cancer.

[4] Wang F, Pang JD, Huang LL, Wang R, Li D, Sun K (2018). Nanoscale polysaccharide derivative as an AEG-1 siRNA carrier for effective osteosarcoma therapy. Int J Nanomedicine.

[5] Harrison DJ, Geller DS, Gill JD (2018). Current and future therapeutic approaches for osteosarcoma. Expert Rev Anticancer Ther 18:39-50. 10.1080/14737140.2018.1413939 29210294

[6] Cheng L, Ke Y, Yu S, Jing J (2016). Co-delivery of doxorubicin and recombinant plasmid pHSP70-Plk1-shRNA by bacterial magnetosomes for osteosarcoma therapy. Int J Nanomedicine.

[7] Doi K, Takeuchi Y (2015). Gene therapy using retrovirus vectors: vector development and biosafety at clinical trials. Uirusu.

[8] Wang H, Jiang Y, Peng H, Chen Y, Zhu P, Huang Y (2015). Recent progress in microRNA delivery for cancer therapy by non-viral synthetic vectors. Adv Drug Deliv Rev.

[9] Wang Y, Li L, Shao N, Hu Z, Chen H, Xu L (2015). Triazine-modified dendrimer for efficient TRAIL gene therapy in osteosarcoma. Acta Biomater.

[10] Peking P, Koller U, Hainzl S, Kitzmueller S, Kocher T, Mayr E (2016). A gene gun-mediated nonviral RNA trans-splicing strategy for Col7a1 repair. Mol Ther Nucleic Acids.

[11] Omata D, Negishi Y, Suzuki R, Oda Y, Endo-Takahashi Y, Maruyama K (2015). Nonviral gene delivery systems by the combination of bubble liposomes and ultrasound. Adv Genet.

[12] Young JL, Dean DA (2015). Electroporation-mediated gene delivery. Adv Genet.

[13] Alsaggar M, Liu D (2015). Physical methods for gene transfer. Adv Genet.

[14] Mah C, Fraites TJ, Zolotukhin I, Song S, Flotte TR, Dobson J (2002). Improved method of recombinant AAV2 delivery for systemic targeted gene therapy. Mol Ther.

[15] Scherer F, Anton M, Schillinger U, Henke J, Bergemann C, Krüger A (2002). Magnetofection: enhancing and targeting gene delivery by magnetic force in vitro and in vivo. Gene Ther.

[16] Plank C, Schillinger U, Scherer F, Bergemann C, Rémy JS, Krötz F (2003). The magnetofection method: using magnetic force to enhance gene delivery. BiolChem.

[17] Fouriki A, Clements MA, Farrow N, Dobson J (2014). Efficient transfection of MG-63 osteoblasts using magnetic nanoparticles and oscillating magnetic fields. J Tissure Eng Regen Med.

[18] Oral O, Cıkım T, Zuvin M, Unal O, Yagci-Acar H, Gozuacik D (2015). Effect of varying magnetic fields on targeted gene delivery of nucleic acid-based molecules. Ann Biomed Eng.

[19] Vainauska D, Kozireva S, Karpovs A, Čistjakovs M, Bariševs M (2012). A novel approach for nucleic acid delivery into cancer cells. Medicina (Kaunas).

[20] Xu Z, Meng K, Cheng J (2015). Highly uniform single-sided portable NMR sensor and its application in assessing the aging level of silicone rubber insulators. Int J Appl Elecrromagn Mech.

[21] He W, He XL, Xu Z, Guo P (2013). The Gram-Schmidt orthogonal data fitting method for the designing of gradient magnetic field of the unilateral NMR[J]. Journal of Chongqing University.

[22] Brissault B, Leborgne C, Guis C, Danos O, Cheradame H, Kichler A (2006). Linear topology confers in vivo gene transfer activity to polyethylenimines. Bioconjug Chem.

[23] Ustinova TM, Yuidin MA, Vengerovich NG, Stepanov AV(2018) Comparative Analysis of Polyethyleneimine Efficiency for Improvement of Plasmid DNA Bioavailability. Bull Exp Biol Med 164:473-477. 10.1007/s10517-018-4015-z 29511894

[24] Namgung R, Singha K, Yu MK, Jon S, Kim YS, Ahn Y (2010). Hybrid superparamagnetic iron oxide nanoparticle-branched polyethylenimine magnetoplexes for gene transfection of vascular endothelial cells. Biomaterials.

[25] Wen M, Li B, Bai W, Li S, Yang X (2014). Application of atomic force microscopy in morphological observation of antisense probe labeled with magnetism. Mol Vis.

[26] Zhou Y, Tang Z, Shi C, Shi S, Qian Z, Zhou S (2012). Polyethylenimine functionalized magnetic nanoparticles as a potential non-viral vector for gene delivery. J Mater Sci Mater Med.

[27] Park W, Yang HN, Ling D, Yim H, Kim KS, Hyeon T (2014). Multi-modal transfection agent based on monodisperse magnetic nanoparticles for stem cell gene delivery and tracking. Biomaterials.

[28] Luo D, Saltzman WM (2000). Enhancement of transfection by physical concentration of DNA at the cell surface. Nat Biotechnol.

[29] Yang HJ, Feng P, Wang L, Li ZC, Ma SP, Wang M (2015). Caveolin-1 mediates gene transfer and cytotoxicity of polyethyleneimine in mammalian cell lines. Mol Cell Biochem.

[30] Zhang G, Gao J, Qian J, Zhang L, Zheng K, Zhong K (2015). Hydroxylated mesoporous nanosilica coated by polyethylenimine coupled with gadolinium and folic acid: a tumor-targeted T(1) magnetic resonance contrast agent and drug delivery system. ACS Appl Mater Interfaces.

[31] Luo X, Peng X, Hou J, Wu S, Shen J, Wang L (2017). Folic acid-functionalized polyethylenimine superparamagnetic iron oxide nanoparticles as theranostic agents for magnetic resonance imaging and PD-L1 siRNA delivery for gastric cancer. Int J Nanomedicine.

[32] Lo YL, Chou HL, Liao ZX, Huang SJ, Ke JH, Liu YS (2015). Chondroitin sulfate-polyethylenimine copolymer-coated superparamagnetic iron oxide nanoparticles as an efficient magneto-gene carrier for microRNA-encoding plasmid DNA delivery. Nanoscale.

[33] Kim d Y, Kwon JS, Lee JH, Jin LM, Kim JH, Kim MS (2015). Effects of the surface charge of stem cell membranes and DNA/polyethyleneimine nanocomplexes on gene transfection efficiency. J Biomed Nanotechnol.

[34] Zhao X, Cui H, Chen W, Wang Y, Cui B, Sun C (2014). Morphology, structure and function characterization of PEI modified magnetic nanoparticles gene delivery system. PLoS One.

[35] Moghimi SM, Symonds P, Murray JC, Hunter AC, Debska G, Szewczyk A (2005). A two-stage poly(ethylenimine)-mediated cytotoxicity: implications for gene transfer/therapy. Mol Ther.

[36] Hall A, Larsen AK, Parhamifar L, Meyle KD, Wu LP, Moghimi SM (2013). High resolution respirometry analysis of polyethylenimine-mediated mitochondrial energy crisis and cellular stress: mitochondrial proton leak and inhibition of the electron transport system. Biochim Biophys Acta.

[37] Remaut K, Oorschot V, Braeckmans K, Klumperman J, De Smedt SC (2014). Lysosomal capturing of cytoplasmic injected nanoparticles by autophagy an additional barrier to non viral gene delivery. J Control Release.

[38] Sonawane ND, Szoka FC, Verkman AS (2003). Chloride accumulation and swelling in endosomes enhances DNA transfer by polyamine-DNA polyplexes. J Biol Chem.

[39] Du J, Zhu W, Yang L, Wu C, Lin B, Wu J (2016). Reduction of polyethylenimine-coated iron oxide nanoparticles induced autophagy and cytotoxicity by lactosylation. Regen Biomater.

[40] Bieber T, Meissner W, Kostin S, Niemann A, Elsasser HP (2002). Intracellular route and transcriptional competence of polyethylenimine-DNA complexes. J Control Release.

[41] Mislick KA, Baldeschwieler JD (1996). Evidence for the role of proteoglycans in cation-mediated gene transfer. Proc Natl Acad Sci U S A.

[42] Bortner CD, Cidlowski JA (1997). Caspase independent/dependent regulation of K(+), cell shrinkage, and mitochondrial membrane potential during lymphocyte apoptosis. J Biol Chem.

[43] Akinc A, Thomas M, Klibanov AM, Langer R (2005). Exploring polyethylenimine-mediated DNA transfection and the proton sponge hypothesis. J Gene Med.

[44] Merdan T, Kunath K, Fischer D, Kopecek J, Kissel T (2002). Intracellular processing of poly(ethylene imine)/ribozyme complexes can be observed in living cells by using confocal laser scanning microscopy and inhibitor experiments. Pharm Res.

[45] Hellmund M, Achazi K, Neumann F, Thota BN, Ma N, Haag R (2015). Systematic adjustment of charge densities and size of polyglycerol amines reduces cytotoxic effects and enhances cellular uptake. Biomater Sci.

[46] Wilhelm C, Billotey C, Roger J, Pons JN, Bacri JC, Gazeau F (2003). Intracellular uptake of anionic supermagnetic nanoparticles as a function of their surface coating. Biomaterials.

[47] Xie L, Jiang Q, He Y, Nie Y, Yue D, Gu Z (2015). Insight into the efficient transfection activity of a designed low aggregated magnetic polyethyleneimine/DNA complex in serum-containing medium and the application in vivo. Biomater Sci.

[48] Huth S, Lausier J, Gersting SW, Rudolph C, Plank C, Welsch U (2004). Insights into the mechanism of magnetofection using PEI-based magnetofectins for gene transfer. J Gene Med.

[49] Ma Y, Zhang Z, Wang X, Xia W, Gu H (2011). Insights into the mechanism of magnetofection using MNPs-PEI/pDNA/free PEI magnetofectins. Int J Pharm.

[50] Ang D, Nguyen QV, Kayal S, Preiser PR, Rawat RS, Ramanujan RV (2011). Insights into the mechanism of magnetic particle assisted gene delivery. Acta Biomater.

[51] Dai Z, Gjetting T, Mattebjerg MA, Wu C, Andresen TL (2011). Elucidating the interplay between DNA-condensing and free polycations in gene transfection through a mechanistic study of linear and branched PEI. Biomaterials.

[52] Itaka K, Harada A, Yamasaki Y, Nakamura K, Kawaguchi H, Kataoka K (2004). In situ single cell observation by fluorescence resonance energy transfer reveals fast intra-cytoplasmic delivery and easy release of plasmid DNA complexed with linear polyethylenimine. J Gene Med.

[53] Lungu CN, Diudea MV, Putz MV, Grudziński IP (2016). Linear and branched PEIs (polyethylenimines) and their property space. Int J Mol Sci.

